# Rhizobacteria regulate colonising *Sitobion avenae* aphid populations through induced host resistance and alter plant volatiles promoting early parasitoid recruitment on barley ( *Hordeum vulgare* )

**DOI:** 10.1002/ps.70783

**Published:** 2026-04-10

**Authors:** Megan E Parker, Angharad M R Gatehouse, Sharon E Zytynska

**Affiliations:** ^1^ Department of Evolution, Ecology and Behaviour Institute of Infection, Veterinary and Ecological Sciences, University of Liverpool Liverpool UK; ^2^ School of Natural and Environmental Sciences, Newcastle Newcastle upon Tyne UK; ^3^ Present address: Department of Earth and Environmental Sciences The University of Manchester Manchester UK

**Keywords:** *Acidovorax*, *Bacillus*, community dynamics, natural enemies, plant volatiles, *Pseudomonas*

## Abstract

**BACKGROUND:**

Soil rhizobacteria can enhance crop resistance to insect herbivores and influence higher trophic interactions, offering potential for sustainable pest management. Aphids are sap‐feeding pests that alter plant physiology, reduce yields and transmit plant viruses. Previous work has shown that inoculation with specific rhizobacterial strains can reduce aphid fitness in short‐term trials. Here, we extend this approach by testing effects across three plant growth stages in glasshouse experiments, monitoring natural colonisation by aphids and their natural enemies in an outdoor pot study, and conducting host‐choice and volatile profile assays.

**RESULTS:**

Under glasshouse conditions, rhizobacterial inoculation consistently suppressed aphid populations, although the magnitude of effects varied with plant variety, bacterial strain and aphid genotype. In our experiments, suppression was strongest when aphids were confined to individual plants, consistent with enhanced host resistance. In outdoor trials, inoculated plants were colonised earlier by parasitoid wasps and harboured more parasitised aphids, independent of aphid density. Volatile assays confirmed that bacterial inoculation altered plant volatile profiles, suggesting a mechanism for enhanced parasitoid attraction. One aphid genotype showed a preference for uninoculated plants in host‐choice assays, indicating potential genotype‐specific avoidance of induced defences.

**CONCLUSION:**

These findings demonstrate that rhizobacteria can influence both bottom‐up and top‐down regulation of aphid populations through plant‐mediated mechanisms. By simultaneously enhancing host resistance and natural enemy attraction, rhizobacterial inoculants offer a promising biological tool for integrated pest management. Understanding context dependency among plant, microbe and insect genotypes will be crucial for translating these interactions into effective and predictable field applications. © 2026 The Author(s). *Pest Management Science* published by John Wiley & Sons Ltd on behalf of Society of Chemical Industry.

## Graphical abstract

Rhizobacterial inoculation of barley reduced *Sitobion avenae* populations by inducing plant resistance and modifying volatiles that attracted parasitoid wasps earlier, enhancing both bottom‐up and top‐down pest control.

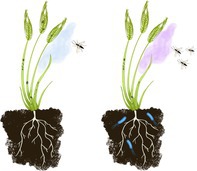

## 1 INTRODUCTION

Interactions between plants, herbivores and their natural enemies are critical drivers of ecosystem dynamics, influencing community structure and function across trophic levels. Among these interactions, rhizobacteria (microorganisms that colonise plant roots) have been shown to modulate plant responses to herbivores through both direct and indirect pathways.^1^ There is growing interest in using rhizobacteria strains or communities as microbial inoculants for sustainable agriculture, helping to increase crop yields and reduce insect pest pressures.^2^ However, most studies are conducted under highly controlled conditions and successful transfer of these results to the field produces variable outcomes.^3^ This approach relies inherently on understanding and exploiting beneficial community and ecological interactions, and thus these interactions must be studied under a community framework.

The rhizosphere is the zone of soil next to plant roots; it is influenced by root exudates and hosts a community of microorganisms that interact closely with the associated plant.^4^ Beneficial rhizobacteria are bacteria living in the rhizosphere that can enhance the health and growth of the associated plant.^5^, ^6^ Rhizobacteria can modulate plant immunity, inducing defences and priming the plant to respond more rapidly to attack by insects or pathogens.^7^, ^8^ Priming refers to an increased capacity for defence within the plant and can involve the modification of histones resulting in faster synthesis of defensive compounds.^9^ A recent meta‐analysis found a general pattern of reduced herbivore fitness on rhizobacteria‐inoculated plants across several plant and insect families.^3^ Rhizobacteria strongly reduced host‐choice and leaf consumption in chewing herbivores, with reduced effects on body size and reproduction. The strongest effects were through reduced reproductive output in sucking herbivores, although few studies have addressed their impact on host‐choice in these insects. Sucking insects like aphids have short generation times and reproduce parthenogenetically, which leads to rapid population expansion, and thus reducing reproductive output is key to population regulation. Natural aphid systems rarely experience population outbreaks, generally attributed to the maintenance of a diversity of ecosystem processes.^10^, ^11^ Yet outbreaks are common in crop fields, leading to the spread of vectored diseases in managed systems and reduced yields.^12^ Ecology‐driven approaches in sustainable agriculture include manipulating the soil microbiome to enhance host‐plant resistance to insect pests.^2^

Rhizobacteria‐induced resistance to insect herbivores is often context dependent, with variation in the strength and even the direction of effect across experimental systems, and controlled pot experiments show stronger significant effects than field trials.^3^, ^13^ Field experiments are more stochastic, not every plant will be colonised at the same time and insects can move freely between treatments rather than being confined to a single pot. Multiple aphid genotypes also exist in the field, which influences population dynamics^14^ driven by genotypic variation in performance and preference for host plants, as well as in interactions with natural enemies.^15^, ^16^, ^17^ Interactions between aphid genotypes and their host plants can be further altered by the presence of rhizobacteria,^18^, ^19^ with cascading effects on parasitoid wasps.^20^ Different crop fields also have different resident soil microbial communities, and the surrounding environment can influence colonisation of herbivores and their natural enemies across the experimental area. All of this results in interactions of variable strength within these communities.^21^, ^22^

In addition to plant defence induction, rhizobacteria may alter plant physiological traits that are associated with attracting the natural enemies of their herbivorous insects.^23^, ^24^, ^25^, ^26^ This provides potential to further reduce herbivore population sizes through top‐down biocontrol. At present there are far fewer studies that consider the cascading impacts of rhizobacteria on natural enemies compared with those measuring direct impacts on herbivores, yet some promising results have been found. For instance, inoculation of *Arabidopsis thaliana* with *Pseudomonas fluorescens* resulted in increased attraction of the parasitoid wasp *Microplitis mediator* to plants infested with *Mamestra brassicae* caterpillars by suppressing the emission of certain plant volatiles.^23^ Inoculation of Brassicaceae with plant‐growth‐promoting *Bacillus* spp. has also been shown to increase parasitism rates of aphids including the cabbage aphid *Brevicoryne brassicae* in the field.^27^, ^28^

In this study, we investigated the long‐term and community‐wide impacts of rhizobacteria inoculation across several experiments (glasshouse, outdoor pot, host‐choice) and undertook an analysis of barley plant volatiles. We hypothesise that rhizobacteria inoculation will decrease aphid population sizes but increase natural enemy colonisation, and rhizobacteria‐mediated aphid suppression will vary across barley growth stages and barley varieties. We also predict that aphids will preferentially colonise control plants compared with inoculated plants.

## 2 MATERIALS AND METHODS

The model system includes varieties of barley (*Hordeum vulgare* L.), and rhizobacteria *Acidovorax radicis* N35, *Bacillus subtilis* B171 and *Pseudomonas simiae* WCS417r. For the outside pot experiment, aphids and natural enemies were allowed to colonise naturally. In the laboratory experiments, our insect herbivore was the cereal aphid *Sitobion avenae* (F.), reared on barley (KWS Curtis) in a temperature‐controlled room set to 18 °C, 65% humidity and a 16:8 h light/dark cycle.

*Acidovorax radicis* N35 bacteria were grown on nutrient agar plates (Difco™ General Purpose Nutrient Broth (BD, Sparks, MD, USA) 8 g/L plus 15 g of agar) at 30 °C for 5 days, then harvested and suspended in 10 mm MgCl_2_ at an optical density at 600 nm (OD_600_) of 2.0. *Bacillus subtilis* B171 and *Pseudomonas simiae* WCS417r were grown separately in nutrient broth for 24 h, centrifuged for 10 min at 4000*g* and the resulting pellet washed three times in 0.9% NaCl (w/v) then spun at 4000*g* for 10 min each time. The cleaned bacterial pellets were resuspended in 10 mm MgCl_2_ and diluted to OD_600_ = 2.0; 10 mm MgCl_2_ was used as the control treatment with no bacteria. The suspensions were used for seed inoculation on the same day, by soaking the seeds in the bacterial suspension (or control solution) for 2 h. In addition, we used an OD_600_ = 1.0 suspension for reinoculation of the glasshouse and outdoor pot experiments. For each experiment, all barley seeds were first washed in 2% sodium hypochlorite for 30 s, then thoroughly rinsed with tap water prior to bacterial inoculation; this was done to remove surface contaminants that may have been present because of the different handling of the barley variety seeds.

### 2.1 Microbe‐induced aphid suppression across barley growth stages

We used a fully factorial experimental design with the three barley varieties [Barbarella (Elsoms), Chevallier (New Heritage Barley Ltd), Irina (KWS)], two bacterial strains [*A. radicis*, *B. subtilis*] and a control, with eight replicates (Supporting Information, Fig. S1(a)). We infested plants with aphids for 2 weeks across three different plant growth stages, with destructive plant sampling at each stage (no repeated measures): (i) early: vegetative growth (3‐week old plants); (ii) middle: tillering (7‐week old plants); and (iii) late: seed development (13‐week old plants). Seeds were surface‐sterilised, inoculated with rhizobacteria by seed soaking and germinated in pots filled with Levington's F1 low‐nutrient potting soil. Plants were reinoculated with bacterial suspension (or control solution) 2 weeks after germination (1 week before the first set of aphid treatments). The experiment was run in a glasshouse (average temperature of 20 °C, with a minimum of 18 °C and occasional peaks of higher temperatures depending on the outside weather, between November 2021 and February 2022).

Two fourth‐instar aphids (*Sitobion avenae*) were transferred to the plants using a paintbrush, at the three experimental time points, and plants were individually covered by a fine mesh and frame. Plant height was measured throughout the experiment, and the numbers of aphids (adults and offspring) were recorded after 14 days. At each time point, plants were harvested following aphid counts and plant dry biomass and root length were measured. For the final time point the number of tillers and seeds per head were counted, and dry biomass and seed mass were measured.

All analyses were completed using R v.4.4.2 in RStudio 2025.09.1. Negative binomial models (glmmTMB) were used to analyse the effect of rhizobacteria inoculation, barley variety and interactions on aphid number after 2 weeks growth. We first combined *A. radicis* and *B. subtilis* treatments to test the effects of the presence and absence of added rhizobacteria, and then analysed them as separated treatments. The first model considered all growth stages together, including growth stage as an additional main effect interacting with barley variety and rhizobacteria treatment. We followed a backwards stepwise approach, first fitting a full model with all interactions and then reducing the model to significant main and interaction effects, with replicate (block) as a random effect.

To account for the high variation in aphid reproductive rate across the plant growth stages, reflecting variable physiological stages and the response of the herbivore (plant growth stage explained up to 87% of variation in aphid numbers), we calculated the relative number of aphids compared with controls (aphid suppression effect). We used the average of each control pot, within each growth stage, because there was no significant effect of block across the experimental space. Linear models with normal error distribution were used to examine effects of bacteria strain (*A. radicis versus B. subtilis*), barley variety and growth stage, including all interactions on the relative number of aphids; also, within plant growth stage as post‐hoc analyses. Plant biomass, as well as tiller number and seed mass for the late growth stage, were examined as response variables using standard mixed‐effect models with rhizobacteria inoculation and variety as main and interacting effects, and replicate (block) as a random effect.

### 2.2 Aphid and natural enemy colonisation of rhizobacteria‐inoculated barley

This experiment was run in an outdoor area within Ness Botanic Gardens (University of Liverpool), enclosed by a large‐herbivore fence (to exclude deer and rabbits). The experimental area was surrounded by diverse trees and other plants, situated in a wider landscape of agricultural fields (May–August 2021). Following a fully factorial randomised complete‐block experimental design (4 barley varieties × 4 bacterial treatments) (Supporting Information, Fig. S1(b)), pots were arranged in 8 replicate blocks of 16 pots. Plants from four spring barley varieties [Barbarella, Chevallier, Irina and an additional variety Sassy (KWS) with UK‐market interest] were inoculated with a control solution (MgCl_2_), *A. radicis*, *B. subtilis* and *P. simiae* (because it has demonstrated impacts for chewing herbivores).

Seeds were inoculated by seed soaking. The barley seeds were germinated in Westland Multi‐Purpose Compost with added John Innes, using seed trays under a polytunnel at Ness Gardens, UK. Seed trays were covered with insect‐protectant netting and kept inside until reaching growth stage 11 (approximately 2 weeks)^29^ then transferred to separate pots (12 cm tall, 10 cm × 10 cm). Plants were reinoculated after transfer to an outside area by adding 7 mL of OD_600_ = 1.0 bacterial suspension or control solution to the soil at the base of the seedlings. Pots were placed into individual 500‐mL plastic containers, to aid watering. Insect mesh was wrapped around the outside of the pots (maintaining an open top) on day 23 to protect plants from slugs and snails.

Aphids and natural enemy abundances were recorded twice weekly from day 14 (18 May) to day 84 (27 July). We identified aphids to species [three main aphid species: *Sitobion avenae, Metopolophium dirhodum* (Walker), *Rhopalosiphum padi* L.], and genus for parasitoid wasps (based on aphid mummy morphology, *Aphidius* spp. and *Praon* spp.). Plant height (from the base of the shoot to the tip of the longest leaf) was measured twice a week until day 65, after which height was measured once a week due to growth rate slowing during tillering. The number of barley tillers/ears was recorded towards the end of the experiment; however, seed yield was not successfully recorded due to overnight herbivory by rabbits despite being fenced in.

For the data analysis, insect abundance data were split into early and late season, representing colonisation before aphid peak abundance, and late season documenting the decline after the peak period. The early season was from 18 May to 29 June during which the barley was tillering and elongating. The late season was from 1 to 27 July during which the barley was heading and ripening. Total numbers of unwinged adults and nymphs, including winged aphids across all species, were combined for total abundances in the early or late season (a measure of aphid pressure). Abundances were then analysed using negative binomial models (glmmTMB), including plot (location) as a random effect, shoot height as a covariate (where appropriate), with rhizobacteria inoculation and barley variety as main effects, and their interaction. We followed a backwards stepwise approach, first fitting a full model with all interactions and then reducing the model to significant main and interaction effects. The total number of natural enemies (including parasitoid wasps, ladybirds, syrphid larvae and spiders) was used to examine effects of season (early and late) on natural enemy abundance, using a negative binomial model. More than half of the natural enemies were parasitoid wasps, and therefore subsequent models (within the early and late seasons) used the number of parasitised aphid ‘mummies’ as the response variable for natural enemy abundance. For the early season we used a Fisher's exact test, because of low count values, to examine the distribution of aphid mummies across rhizobacteria treatments and barley varieties. In the late season, we used negative binomial models including total unwinged and winged aphids as covariates to account for variable aphid colony sizes across plant replicates. For the plant data, in the early season we examined the effects of rhizobacterial treatment and barley variety on plant height, leaf number and tiller number using linear mixed effects models with normal error distributions, with aphid number as a covariate and plot location as a random effect. For the late season, we used plant height and number of tillers with seeds (yield) as response variables.

### 2.3 Rhizobacteria inoculation effects on aphid host‐choice

To explore the effects of bacterial inoculation on aphid host‐plant choice, an aphid host‐choice experiment was conducted, using Irina barley and *A. radicis* and *B. subtilis*; we selected these for further exploration based on the responsiveness of this barley to these bacterial strains in the previous experiments. We infested plants with two different aphid genotypes (individually, i.e. no mixed genotype treatments). These aphids were collected from an experimental field at Stockbridge Technology Centre, near York, UK in 2022: a green colour morph belongs to the common UK clone SA‐1, while a pink coloured morph is a novel clone not associated with any common UK clone and genetically distinct from green SA‐1 (data not shown).

Irina seeds were surface‐sterilised and germinated in seed trays (Levington's F1 low‐nutrient soil) and kept in a climate chamber (20 °C 16:8 h light/dark photoperiod and 65% humidity). After 1 week, seedlings were transplanted individually into small polythene bags to keep soil and roots separate and prevent cross‐contamination of inoculants. We arranged three plants per pot with one of each rhizobacteria inoculation (three‐way choice: *A. radicis, B. subtilis* and control) or all seedlings with the same rhizobacteria treatment or control (no choice), with a layer of sand on top to allow ease of aphid movement (Supporting Information, Fig. S1(c)).

Two days after transplantation, seedlings were each infested with 3 fourth‐instar aphids (green or pink genotype) placed on a 25‐mm diameter paper circle in the centre of each pot, which was then covered with a mesh cage. All aphids moulted into adults within the first 24 h of being added to the pots and all pots produced nymphs by day 4. Aphid adults and nymphs were counted for each individual barley plant along with seedling height on days 1, 4, 7, 11 and 14.

Aphid final population size per pot was analysed with negative binomial models (glmmTMB), using shelf (location) as a block effect, total shoot height as a covariate, with aphid genotype and choice (choice or no choice) as main effects and their interaction. The same approach was used for no‐choice pot subset, with rhizobacteria treatment and aphid genotype as main and interacting effects, to examine pot‐level suppression effects of rhizobacteria treatment. Proportional data (per plant) were normalised with an arcsine square root transformation and analysed with linear mixed‐effect models, using aphid genotype and rhizobacteria treatment as main effects; separated for choice and no‐choice pots. Time was included as a main effect where relevant, otherwise it was included as a random effect along with shelf location in the growth chamber. Owing to differences across aphid genotypes, we further analysed these individually for post‐hoc analysis within choice pots.

### 2.4 Headspace collection of barley volatiles

Irina and Firefoxx seeds were germinated in seed trays (Levington's F1 low‐nutrient soil) and kept in a climate chamber (20 °C 16:8 h light/dark photoperiod and 65% humidity). After 1 week, individual seedlings were transferred to 50‐mL falcon tubes and a sodium alginate bead containing either *A. radicis*, *B. subtilis* B171 or MgCl_2_ (control) was added to the soil next to the base of the seedling and covered, following optimised methods.^30^ After 48 h, 10 third‐instar aphids (5 green and 5 pink) were added to half of the replicates and all plants were covered with a fine nylon mesh cage. There were six replicates per treatment. After a futher 48 h, all aphids and cages were removed, the seedlings were covered with transparent bags (31 cm × 30 cm, Toppits® Bratschlauch, Melitta Group, Minden, Germany, PET film) secured at each end and the soil was also covered with aluminium foil. A Tenax TA (35/60) inert coated probe (Markes) was inserted into the bag and positioned next to the seedlings using steel wire, the bag was then sealed using nylon string. After 24 h the probes were collected, sealed with gold caps and stored at −80 °C until analysis on an Agilent 7250 GC QTOF‐MS with a HP‐5MS (30 m x 0.25 mm x 0.25 μm) column connected to a Markes TD 100xr. The probes were dry purged with nitrogen for 5 min at 50 mL min^−1^ before loading onto the Markes system to removed adsorbed water. There was a 1‐min probe pre‐purge with 150 °C flow path temperature, 5‐min single stage desorption at 280 °C and trap desorption for 3 min at 300 °C. The trap flow was 50 mL min^−1^ and the split flow was 2 mL min^−1^. The gas chromatography–mass spectrometer (GC–MS) used a universal split/splitless single taper wool liner with an inlet temperature of 150 °C. Samples were analysed with a helium flow rate of 1 mL min^−1^. The start temperature was 40 °C with a ramp speed of 6 °C min^−1^ and no hold time. Stage one used a temperature of 170 °C with a ramp speed of 15 °C min^−1^ and no hold time. The final temperature was 190 °C with no hold time. The post‐run temperature was 280 °C with a hold time of 2 min and the transfer line temperature 300 °C. Each run took 23 min, the acquisition rate was 10 Hz and the mass range was 50–600 *m*/*z*. In addition to the sample probes, we ran lab air blanks, Tenax probe blanks and a retention index loaded probe.

Data were processed in MSDIAL v.4.93 and peak annotation was performed using a combination of an in‐house spectral reference library and purchased Fiehn, NIST20 and GOLM libraries. In cases in which spectral similarity was <70%, the nearest hit was reported. Blank subtraction was performed on the data using blank probes, lab air, climate chamber and bag‐only probes. Data were normalised according to internal standards, scaled and transformed by log_10_. Features were assigned a metabolite number to avoid bias during statistical analysis and to allow the inclusion of unknowns. Analysis of the normalised and transformed data was performed in R (v.4.1.0) using RStudio (v.2024 12.0). Variation in volatile profiles was examined using non‐metric multidimensional scaling (NMDS) and Permutational Multivariate Analysis of Variance (PERMANOVA) based on Bray–Curtis dissimilarities, alongside redundancy analysis (RDA). Significance of experimental factors on the RDA‐derived sample scores was assessed using linear models.

## 3 RESULTS

### 3.1 Microbe‐induced aphid suppression across barley growth stages

The plant growth stage at which aphids were added to the plants significantly affected final aphid population size (*X* ^2^_2_ = 105.33, *P <* 0.001). Aphid populations grew largest on plants during the early growth stage with 145 ± 6 (mean ± SE) aphids, compared with during the tillering stage (108 ± 6), with even fewer on plants infested during seed development (68 ± 6). Averaged across all growth stages, aphid populations varied with barley variety (*X* ^2^_2_ = 6.77, *P* = 0.034), with larger populations on Irina and Chevallier, and lowest on Barbarella.

**Figure 1:**
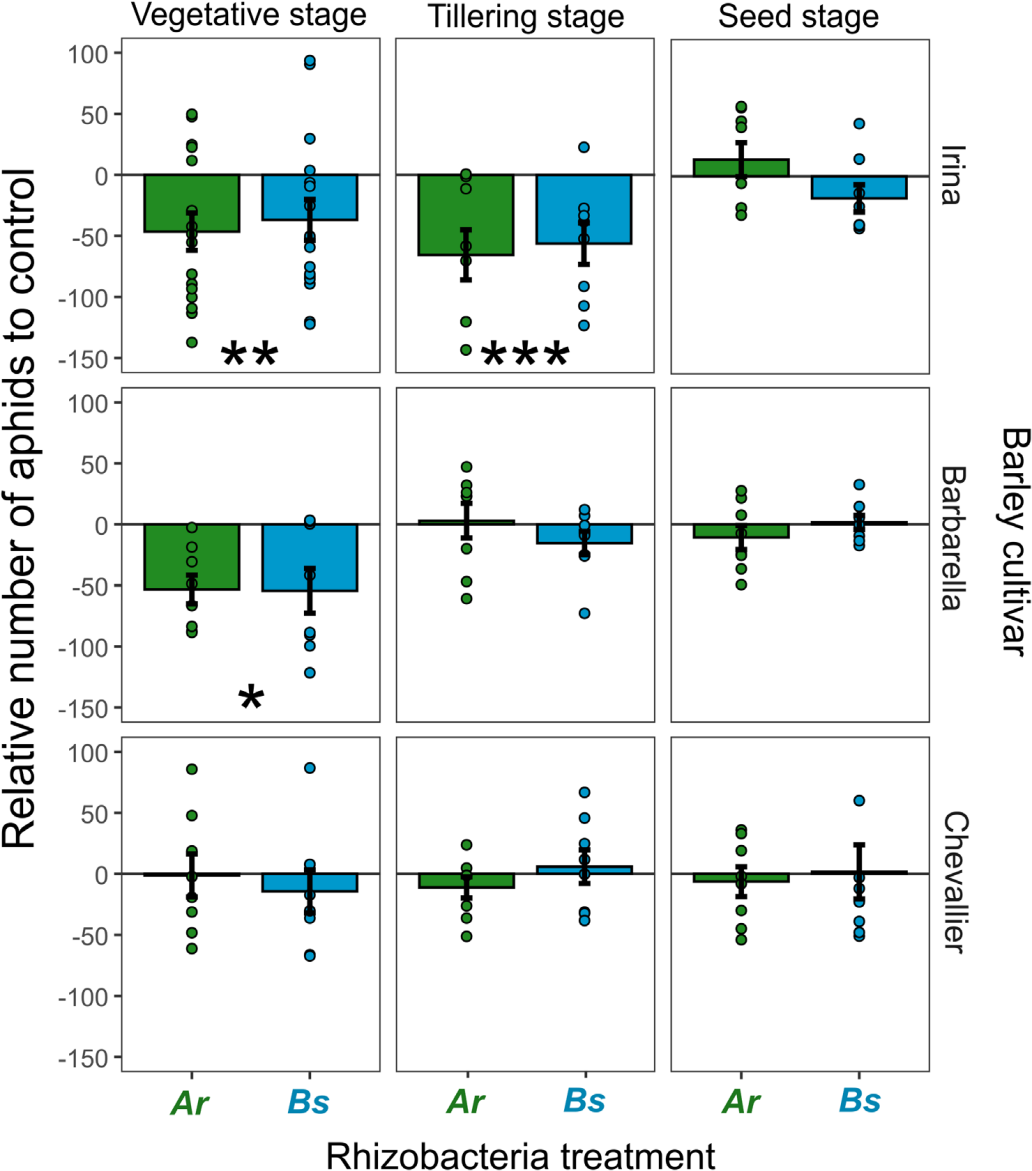
Relative aphid abundance on bacterial‐inoculated plants compared with control plants across the three plant growth stages and three barley varieties. Ar, *Acidovorax radicis* (green); Bs, *Bacillus subtilis* (blue). Points represent individual observations (replicates), error bars show ±1SE. *P < 0.05, **P < 0.01, ***P < 0.001.

Rhizobacteria inoculation of plant roots significantly reduced aboveground aphid numbers, averaged across all growth stages (*X* ^2^_1_ = 7.69, *P* = 0.006). By calculating the relative number of aphids on rhizobacteria‐inoculated compared with control plants, we showed that there was no difference in magnitude of effect across the two rhizobacteria strains (*X* ^2^_1_ = 0.04, *P* = 0.984). However, aphid suppression was weaker in the later growth stages (*X* ^2^_2_ = 6.96, *P* = 0.006), varied across barley varieties (*X* ^2^_2_ = 6.96, *P* = 0.006) and these effects depended on both variety and growth stage (*X* ^2^_4_ = 3.49, *P* = 0.009) (Fig. 1).

At the early vegetative growth stage, we observed aphid suppression by both rhizobacteria (rhizobacteria presence: *X* ^2^_1_ = 8.07, *P* = 0.004). When comparing relative effect sizes, we observed suppression effects for the two modern barley varieties Barbarella (*z* = −2.36, *P* = 0.022) and Irina (*z* = −2.00, *P* = 0.005), but not for the heritage barley variety, Chevallier (*t* = −0.57, *P* = 0.574). By the tillering stage, there was a weaker effect of rhizobacteria inoculation on aphid number (rhizobacteria presence: *X* ^2^_1_ = 3.34, *P* = 0.068) than at the vegetative stage, with significant suppression only observed on Irina (*z* = −4.07, *P<*  0.001). By the seed stage, when aphid reproductive output was lowest, we observed no significant aphid suppression across any barley variety.

Although we observed no significant effect of rhizobacteria inoculation on shoot growth (*F*_2,158_ = 1.71, *P* = 0.184) or root growth (*F*_2,160_ = 0.79, *P* = 0.456), rhizobacteria inoculation significantly affected tiller number in the seed‐producing stage (*F*_2,65_ = 3.80, *P* = 0.027). This was driven by rhizobacteria inoculation with *A. radicis* resulting in higher numbers of tillers than in *B. subtilis‐*inoculated plants (*z* = −2.68, *P* = 0.009). Barbarella produced the greatest seed mass (11.4 ± 0.41 g), followed by Irina (8.24 ± 0.57 g); Chevallier produced the lowest yield (5.72 ± 0.43 g) (*F*_2,67_ = 35.37, *P <* 0.001; Supporting Information, Fig. S2(a)). Chevallier barley plants had a larger biomass than the other varieties (*F*_2,67_ = 3.63, *P* = 0.032; Supporting Information, Fig. S2(b)). No effect of rhizobacterial inoculation seed mass (*F*_2,60_ = 0.12, *P* = 0.885) was observed.

### 3.2 Aphid and natural enemy colonisation of rhizobacteria‐inoculated barley

Aphids colonised plants within 7 days of being transferred outside. The three most abundant aphid species observed were English grain aphid (*Sitobion avenae*), rose grain aphid (*M. dirhodum*) and bird cherry‐oat aphid (*Rhopalosiphum padi*) (Supporting Information, Fig. S3). Aphids colonised from the southeast, leading to a strong gradient across the experimental area for winged aphid colonisation (*X* ^2^_1_ = 36.06, *P <* 0.001) with a reduced impact on unwinged aphids (*X* ^2^_1_ = 8.08, *P* = 0.004) and a non‐significant effect on natural enemies (*X* ^2^_1_ = 3.27, *P* = 0.071). We accounted for this environmental gradient by including block in the models. The overall population of aphids increased steadily over the early half of the season and peaked at approximately day 58 (29 June) for most of the treatments, then decreased again towards the end of the season (Supporting Information, Fig. S3).

#### 3.2.1 Seasonal effects

The number of unwinged aphids and natural enemies differed significantly across the early and late season. We counted 5557 unwinged aphids in the early season and 7697 in the late season (*X* ^2^_1_ = 0.021, *P* = 0.021, also see Supporting Information, Fig. S3), and there were more natural enemies in the late season (*n* = 292, *n* = 199 parasitised aphid mummies, *n* = 11 adult parasitoid wasps, *n* = 6 ladybird adults, *n* = 18 ladybird larvae, *n* = 16 Syrphid larvae, *n* = 42 spiders) than early (*n* = 153, *n* = 27 parasitised aphid mummies, *n* = 16 adult parasitoid wasps, *n* = 3 ladybird adults, *n* = 17 ladybird larvae, *n* = 21 Syrphid larvae, *n* = 69 spiders) (*F*_1,243_ = 77.32, *P <* 0.001). The number of winged aphids did not vary between early and late season (early *n* = 245, late *n* = 232; *X* ^2^_1_ = 0.11, *P* = 0.745).

**Figure 2:**
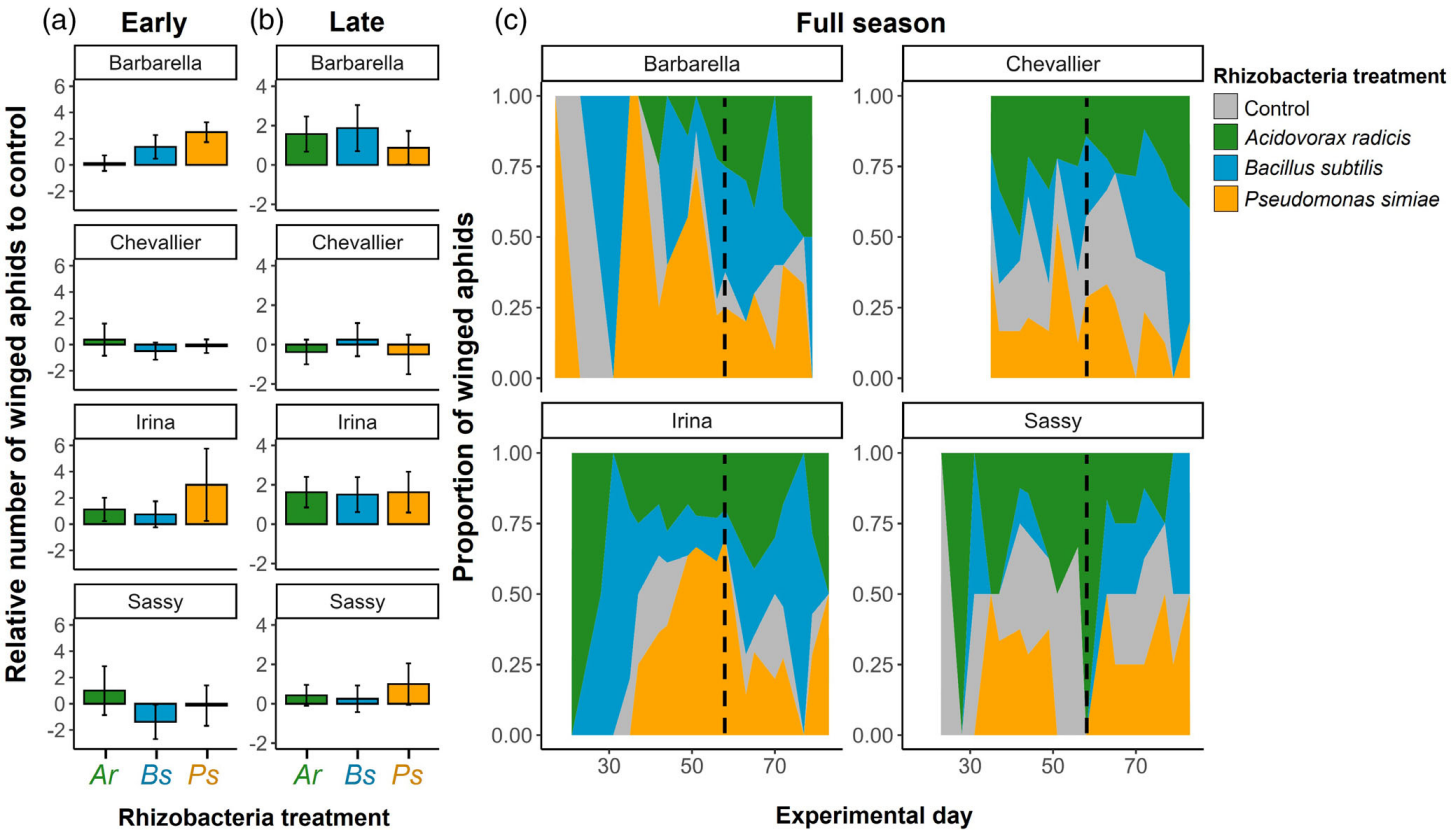
Abundance of winged aphids (all species) on barley plants. Relative means of aphids compared to control plants (within block comparisons) for early (a) and late (b) season. Error bars show ±1 SE. (c) Distribution of winged aphids across rhizobacteria treatments for each barley variety and across the experimental duration. Control, control treatment (grey); Ar, *Acidovorax radicis* (green); Bs, *Bacillus subtilis* (blue); Ps, *Pseudomonas simiae* (yellow). Dashed line shows the split between early and late season for each barley variety. Empty space indicates no aphids found on plants of this variety.

**Figure 3:**
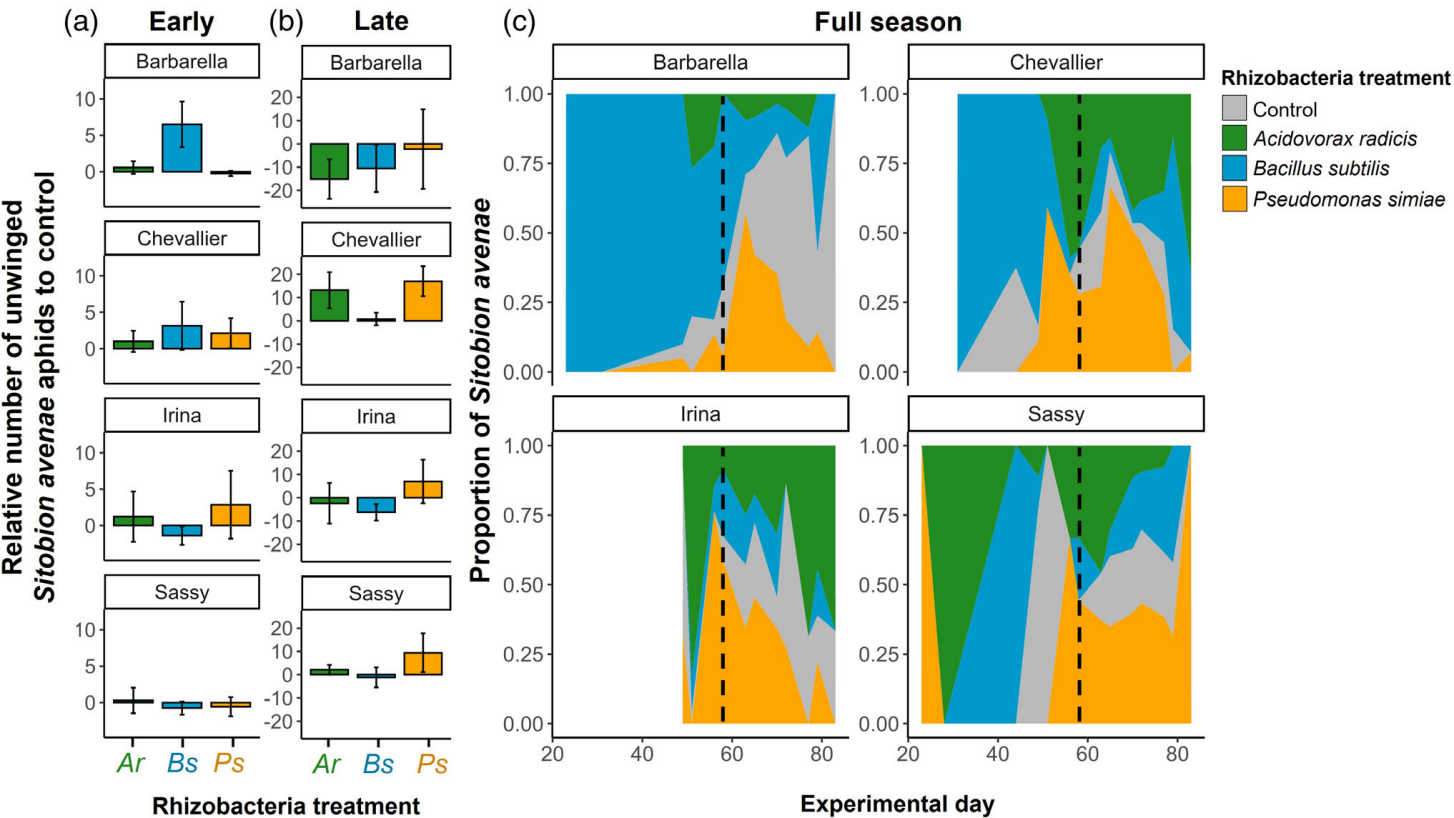
Abundance of unwinged *Sitobion avenae* aphids on barley plants. Relative means of aphids compared with control plants (within block comparisons) for early (a) and late (b) season. Error bars show ±1SE. (c) Distribution of unwinged *S. avenae* aphids across rhizobacteria treatments for each barley variety and across the experimental duration. Control, control treatment (grey); Ar, *Acidovorax radicis* (green); Bs, *Bacillus subtilis* (blue); Ps, *Pseudomonas simiae* (yellow). Dashed line shows split between early and late season for each barley variety. Empty space indicates no aphids found on plants of this variety.

For winged aphids, there were more aphids on rhizobacteria‐inoculated plants (presence/absence: *X* ^2^_1_ = 7.53, *P* = 0.006) across the whole season. Within inoculation treatments, rhizobacteria strain effects were dependent on season (*X* ^2^_2_ = 6.00, *P* = 0.049), indicating strain‐specific effects (Fig. 2). Across all aphid species, the simple presence of rhizobacteria had some influence on the abundance of unwinged aphids dependent on season (*X* ^2^_1_ = 3.24, *P* = 0.072). When analysed by aphid species, the abundance of *S. avenae* aphids was influenced by rhizobacteria treatment dependent on season (*X* ^2^_3_ = 11.50, *P* = 0.009) and on barley variety (*X* ^2^_9_ = 19.44, *P* = 0.022) (Fig. 3); *R. padi* aphid abundance was similarly influenced by rhizobacteria treatment dependent on season, and across barley variety as a three‐way interaction (*X* ^2^_9_ = 21.74, *P* = 0.010; Supporting Information, Fig. S4); *M. dirhodum* aphid abundance did not vary across the season but we detected some effect of rhizobacteria treatment dependent on barley variety (*X* ^2^_9_ = 16.61, *P* = 0.055; Supporting Information, Fig. S5). The abundance of natural enemies also differed across the season dependent on rhizobacteria treatment (*X* ^2^_9_ = 10.50, *P* = 0.015). Based on this, we now explore these in more detail, separately for the early (before day 58, up to 29 June) and late season (after day 58, 30 June onwards).

#### 3.2.2 Winged aphids

In the early part of the season, rhizobacteria treatment affected winged aphid number on plants (*X* ^2^_3_ = 9.16, *P* = 0.027), with some evidence that the effect of rhizobacteria treatment was dependent on barley variety (*X* ^2^_9_ = 15.29, *P* = 0.083) (Fig. 2) but not mediated by plant growth differences (*X* ^2^_1_ = 1.43, *P* = 0.232). In particular, more winged aphids were observed on *P. simiae*‐inoculated plants (*z* = 2.18, *P* = 0.029) than others (Fig. 2). In the latter part of the season, more winged aphids again colonised inoculated plants (*X* ^2^_1_ = 5.02, *P* = 0.025) but the effect size no longer varied among rhizobacteria strains (*X* ^2^_2_ = 0.35, *P* = 0.838) (Fig. 2).

#### 3.2.3 Unwinged aphids

For unwinged aphids (all aphid species), we observed significant variation in aphid abundance in the early season as a result of higher winged aphid abundance (*X* ^2^_1_ = 9.93, *P* = 0.002), as well as across rhizobacteria treatments dependent on barley variety (*X* ^2^_9_ = 30.44, *P <* 0.001); these interaction effects were variable across experimental area (block interaction: *X* ^2^_9_ = 33.10, *P <* 0.001), highlighting variability in aphid population dynamics. In the late season, we observed similar patterns with winged aphid abundance driving unwinged aphid numbers (*X* ^2^_1_ = 20.86, *P <* 0.001) and continued effects of rhizobacteria treatment dependent on barley variety (*X* ^2^_9_ = 19.24, *P* = 0.023). We explore further by aphid species as the patterns varied.

*Sitobion avenae* aphids were most abundant in the late season (*n* = 1547) compared to the early season (*n* = 267) (Fig. 3), and *S. avenae* abundance was positively associated with winged aphid abundance (early: *X* ^2^_1_ = 5.39, *P* = 0.020; late: *X* ^2^_1_ = 4.55, *P* = 0.033). In the early season, aphid abundance was significantly influenced by rhizobacteria treatment (*X* ^2^_3_ = 9.05, *P* = 0.029), with more *S. avenae* aphids observed on *B. subtilis* plants (Fig. 3(a),(c)). In the later season, there was a significant interaction between rhizobacteria treatment and barley (*X* ^2^_6_ = 17.54, *P* = 0.041), this was driven by different treatment effects (Fig. 3(b),(c)). In particular, there were fewer aphids on *A. radicis*‐inoculated Barbarella compared with other barley varieties (*z* = −2.30, *P* = 0.021), and fewer aphids on *B. subtilis*‐inoculated Chevallier compared with other rhizobacteria strains (*z* = 2.53, *P* = 0.011).

For *R. padi* aphids, variable effects of rhizobacteria across the early and late season, dependent on barley variety (*X* ^2^_9_ = 21.74, *P* = 0.010), were driven in part by reduced colonisation of *P. simiae* Barbarella plants in the later season (*z* = −1.98, *P* = 0.047) and increased colonisation of control‐inoculated plants across barley varieties (*z* = 2.17, *P* = 0.030) (Supporting Information, Fig. S4). Within seasons we did not detect any further variation explained by rhizobacteria treatment or barley variety. There was also no significant association between *R. padi* aphid number and the number of winged aphids (*X* ^2^_1_ = 1.38, *P* = 0.112).

For *M. dirhodum* aphids, there was a significant positive association with the number of winged aphids (early: *X* ^2^_1_ = 10.01, *P* = 0.002, late: *X* ^2^_1_ = 8.47, *P* = 0.004). Exploring the interaction between rhizobacteria and barley variety across both seasons (*X* ^2^_9_ = 16.61, *P* = 0.055) (Supporting Information, Fig. S5), highlights that *M. dirhodum* aphids were rarely observed on Sassy plants inoculated with *B. subtilis* (*z* = −2.10, *P* = 0.036), but were abundant on other varieties. No other significant effects were observed within season.

#### 3.2.4 Natural enemies – parasitoid wasps

**Figure 4:**
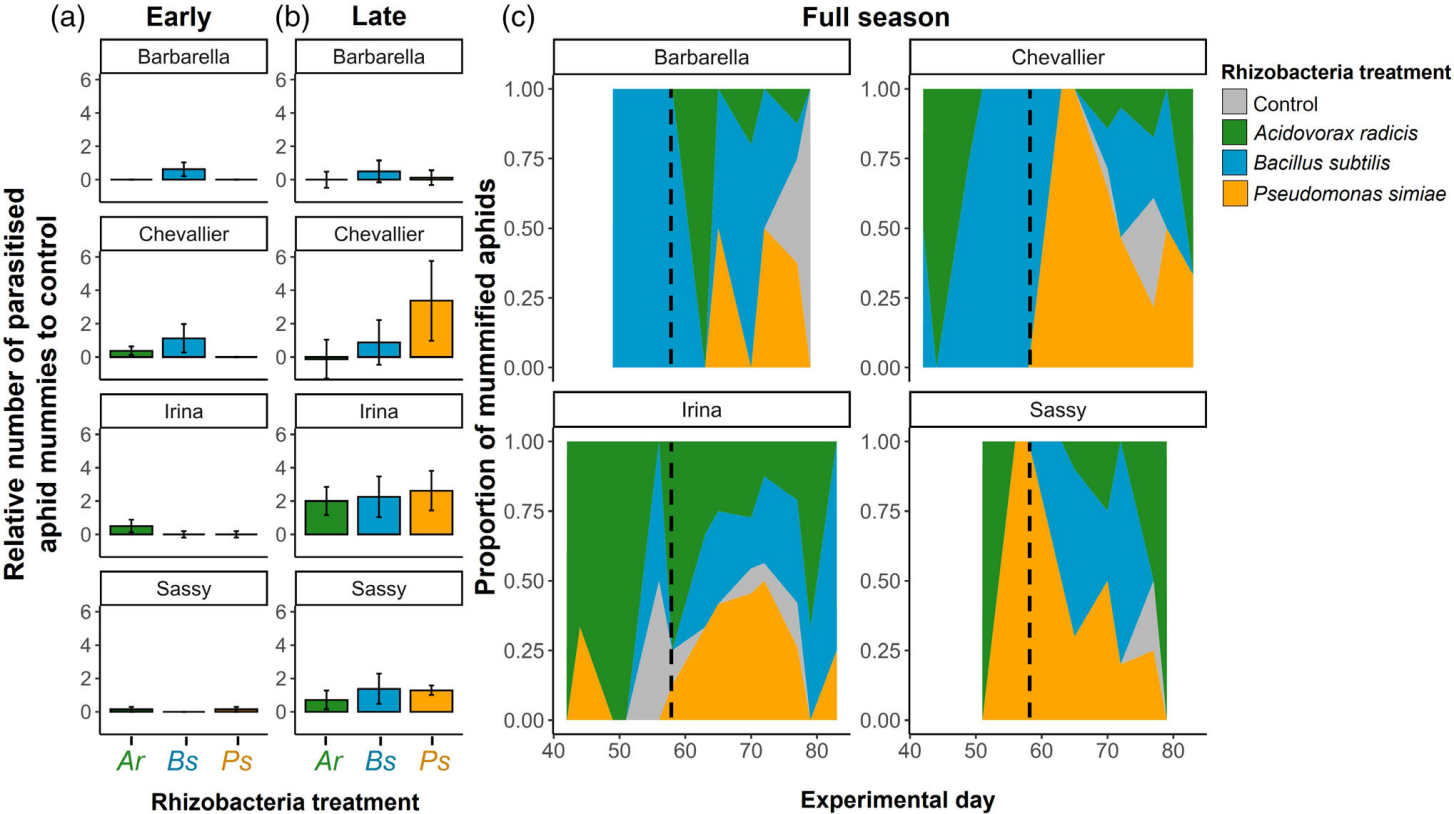
Abundance of parasitoid wasp aphid ‘mummies’ on barley plants. Relative means of parasitised aphid mummies compared with control plants (within block comparisons) for early (a) and late (b) season. Error bars show ±1SE. (c) Distribution of aphid mummies across rhizobacteria treatments for each barley variety and across the experimental duration. Control, control treatment (grey); Ar, *Acidovorax radicis* (green); Bs, *Bacillus subtilis* (blue); Ps, *Pseudomonas simiae* (yellow). Dashed line shows split between early and late season for each barley variety. Empty space indicates no aphid mummies found on plants of this variety.

The most relevant natural enemies for aphids in this experiment were parasitoid wasps. Adult parasitoid wasps and parasitised aphid mummies were first observed on day 42, approximately 2 weeks before aphid peak population. In the early season, as few as 27 parasitised aphid mummies were observed on 15 plants, with significant variation among rhizobacteria treatments and barley varieties (Fisher's exact test, *P* = 0.003) (Fig. 4(a)). Only one parasitised aphid was found on uninoculated control plants, six on each of *B. subtilis* (Barbarella and Chevallier) and *A. radicis* (Irina and Chevallier) plants, and two on *P. simiae*‐inoculated plants (Fig. 4(a), (c)). Thus, of the 27 parasitised aphids observed before the peak of aphid populations only one was observed on a uninoculated control plant.

By the late season, 199 parasitised aphids were counted and these were again found predominantly on inoculated plants (rhizobacteria presence: *X* ^2^_1_ = 6.95, *P* = 0.008). As expected, there were more parasitised aphids on plants with larger aphid populations (*X* ^2^_1_ = 7.63, *P* = 0.006). Within rhizobacteria inoculation treatments, we again found significant differences in aphid parasitism on plants (*X* ^2^_3_ = 9.78, *P* = 0.020) (Fig. 4(b),(c)), and variation across barley varieties (*X* ^2^_3_ = 13.45, *P* = 0.004) (Fig. 4(b),(c)), but these variables did not significantly interact (*X* ^2^_9_ = 6.64, *P* = 0.674). In general, we observed more parasitised aphids on *B. subtilis* (*z* = 2.47, *P* = 0.014) and *P. simiae* (*z* = 2.92, *P* = 0.004) inoculated plants, and on Chevallier (*z* = 2.09, *P* = 0.037) and Irina (z = 1.76, *P* = 0.079) varieties, when controlling for aphid population size.

#### 3.2.5 Plant growth and yield

At the end of the early season, there was significant height variation among barley varieties (*X* ^2^_3_ = 27.32, *P <* 0.001); Chevallier (36.4 ± 1.16 cm) and Barbarella (35.4 ± 0.96 cm) plants were tallest, with Irina (31.4 ± 1.05 cm) and Sassy (31.8 ± 1.23 cm) plants smaller. Rhizobacteria treatment altered plant height (*X* ^2^_3_ = 8.19, *P* = 0.042), independent of barley variety. Plants inoculated with *B. subtilis* were on average 8% smaller than control, *A. radicis* or *P. simiae*‐inoculated plants (*t* = 2.75, *P* = 0.007). There were no other significant effects on plant leaf number or tiller number in the early season.

In the late season, there was no longer any effect of rhizobacteria on plant height (*X* ^2^_3_ = 3.43, *P* = 0.331), but there were strong differences across barley varieties (*X* ^2^_3_ = 153.88, *P <* 0.001) with Chevallier plants growing 30% taller than the other varieties (Chevallier 79.5 ± 2.92 cm, average others 59.3 ± 0.72 cm). Chevallier produced the fewest seed heads (6.00 ± 0.45) compared with other varieties (average others 10.1 ± 0.27) (*X* ^2^_3_ = 98.78, *P <* 0.001), with no effect of rhizobacteria treatment (*X* ^2^_3_ = 1.94, *P* = 0.586) (Supporting Information, Fig. S6).

### 3.3 Microbial inoculation effects on aphid host‐choice

There was no difference in the total number of aphids (summed across all three plants) between choice and no‐choice pots at day 14 (*X* ^2^_1_ = 0.63, *P* = 0.428), and no difference in final aphid population size for green and pink aphid lines (*X* ^2^_1_ = 0.02, *P* = 0.895). Thus, any differences in the distribution of aphids across plants was not due to variable density effects.

#### 3.3.1 No‐choice pots

**Figure 5:**
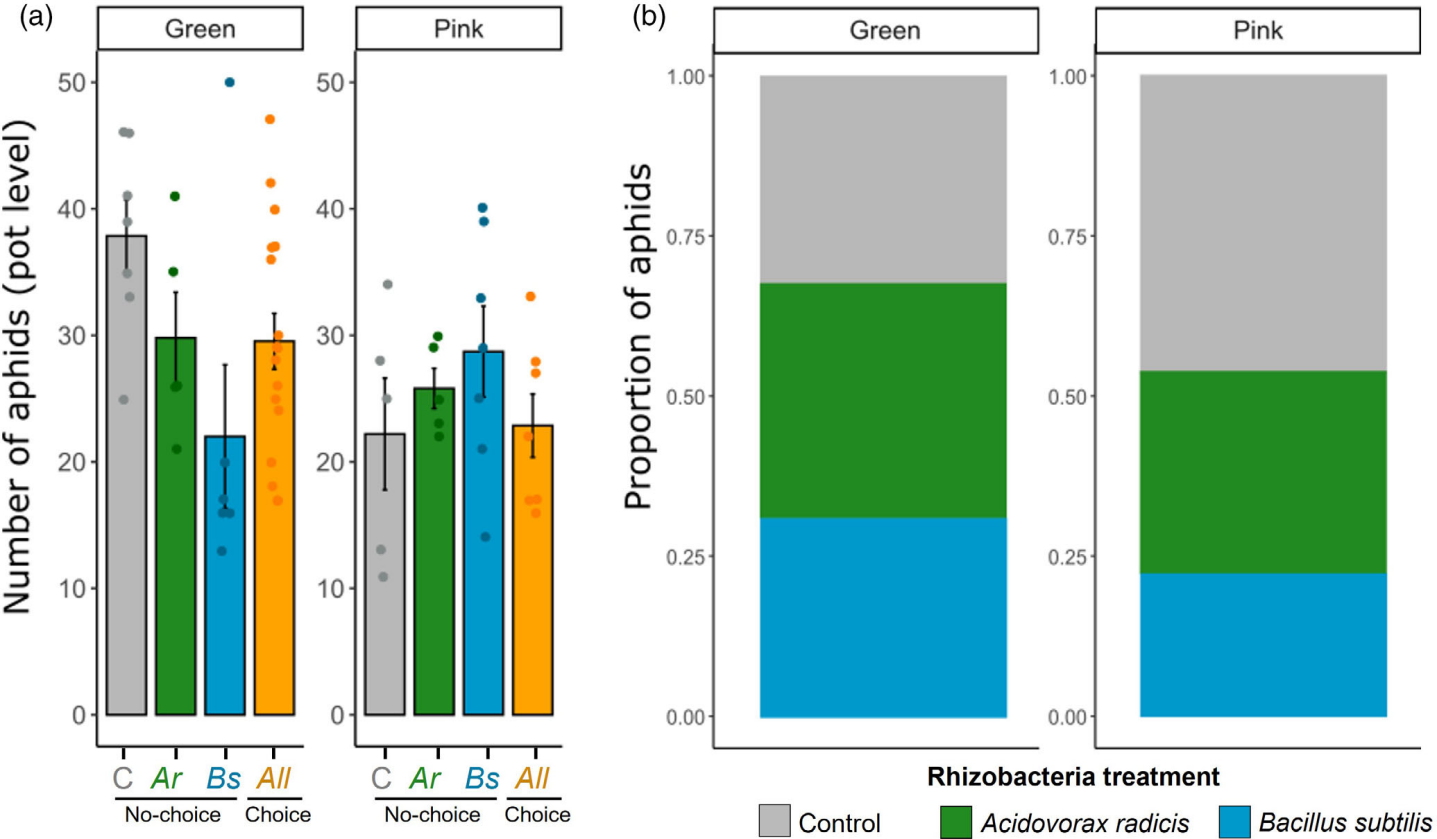
Aphid host‐choice across rhizobacteria‐inoculated and control plants. (a) Proportion of aphids in each pot by treatment on Irina barley inoculated with *Acidovorax radicis* (green) or *Bacillus subtilis* (blue) or control solution (grey) across full experiment (no significant variation in aphid distribution over time). (b) Final aphid population size (adults and offspring) at the pot level. For no‐choice pots, the three plants were inoculated with the same treatment: control treatment (grey); Ar, *Acidovorax radicis* (green); Bs, *Bacillus subtilis* (blue). For the choice pots (orange), each plant had a different rhizobacteria treatment. Bars show means and error bars ±1SE.

In no‐choice pots, where all three plants were inoculated with the same treatment (*A. radicis* or *B. subtilis*) or control pots, the different aphid lines varied in their response across rhizobacteria treatments as examined by total aphid number at the pot level (*X* ^2^_2_ = 8.69, *P* = 0.013) (Fig. 5(a)). Significant aphid suppression was only observed for green aphids, and specifically for pots inoculated with *B. subtilis* (*z* = −2.90, *P* = 0.004); with high variation across replicates noted under this experimental design. Both adult aphids and offspring (produced over the experimental period) were evenly spread across the three plants in the no‐choice pots (adult: *X* ^2^_2_ = 1.35, *P* = 0.509; offspring: *X* ^2^_2_ = 3.84, *P* = 0.146), and their distribution did not change over time (adult: *X* ^2^_4_ = 0.29, *P* = 0.991; offspring: *X* ^2^_4_ = 1.04, *P* = 0.904).

#### 3.3.2 Choice pots

For choice pots, with the three plants comprising different rhizobacteria treatments (control, *A. radicis* or *B. subtilis*), we focus on the proportion of aphids on each plant within pot. The distribution of aphids was dependent on both aphid genotype and rhizobacteria treatment (adults: *X* ^2^_2_ = 7.96, *P* = 0.019; offspring: *X* ^2^_2_ = 8.18, *P* = 0.017), this pattern was consistent over the time points (adult: *X* ^2^_4_ = 0.10, *P* = 0.998; offspring: *X* ^2^_4_ = 0.43, *P* = 0.979) (Supporting Information, Fig. S7).

Pink aphids were found in greater abundance on control plants (adults: *X* ^2^_2_ = 15.33, *P <* 0.001; offspring: *X* ^2^_2_ = 5.79, *P* = 0.055), with significantly reduced numbers of adults and offspring on *A. radicis* (adults: *t* = −2.65, *P* = 0.009; offspring: *t* = −2.25, *P* = 0.025) and *B. subtilis* (adults: *t* = −1.91, *P* = 0.057; offspring: *t* = −2.43, *P* = 0.016) plants (Fig. 5(b)). By contrast, there was no influence of rhizobacteria treatment on the distribution of green aphids within the choice pots (adults: *X* ^2^_2_ = 0.48, *P* = 0.787; offspring: *X* ^2^_2_ = 3.49, *P* = 0.174) (Fig. 5(b)). There was high variability in population sizes across replicates, pink aphids generally reached average population size of 25 ± 1.6 (mean ± SE), whereas green aphids reached average population size of 30 ± 1.8.

### 3.4 Headspace analysis of barley volatiles

**Figure 6:**
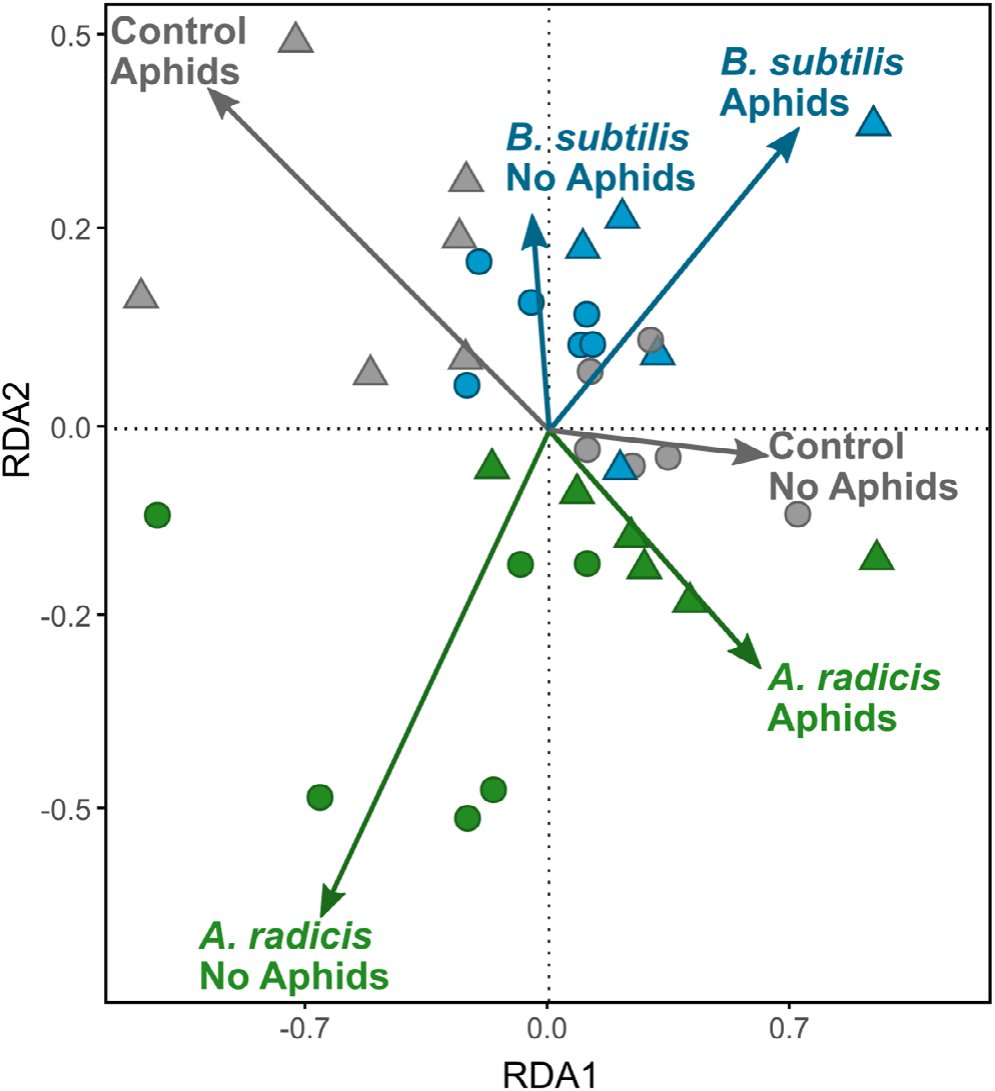
Redundancy analysis of headspace volatile profiles of Irina barley. Across rhizobacteria treatments [*Acidovorax radicis* (green), *Bacillus subtilis* (blue) or control (grey)] and aphids [no aphids (circle) or with aphids (triangles)] for Irina barley. Points represent samples, and arrows show explanatory variables; their direction and length indicate their influence.

We performed untargeted analysis of barley headspace and identified 308 metabolites to examine profile differences across bacterial and aphid treatments for Irina barley plants. We observed significant variation in metabolome profile due to bacterial inoculation (RDA2: *F*_1,29_ = 31.79, *P* < 0.001) (Fig. 6), aphid presence (RDA2: *F*_1,29_ = 13.35, *P* = 0.001) (Fig. 6), and an interaction between rhizobacteria and aphid treatments (RDA1: *F*_2,29_ = 16.57, *P* < 0.001) (Fig. 6). NMDS ordination and PERMANOVA analyses based on Bray–Curtis dissimilarities did not detect significant treatment effects on overall volatile profiles (bacteria: *R* ^2^ = 0.045, *P* = 0.77; aphids: *R* ^2^ = 0.032, *P* = 0.81; bacteria × aphids: *R* ^2^ = 0.046, *P* = 0.78), indicating that treatment effects occur primarily at the level of individual compounds rather than broad changes in blend composition. Several individual compounds were identified as differing across main and interaction effects; however, we do not present data on chemical identification, because these analyses focus on relative differences and patterns of variation among treatments. In general, *A. radicis*‐inoculated plants showed stronger differentiation compared with control plants than did *B. subtilis*‐inoculated plants. Interestingly, aphid‐induced headspace volatiles showed contrasting effects across control and rhizobacteria‐inoculated plants.

## 4 DISCUSSION

We combined controlled‐environment and outdoor pot experiments to test how rhizobacteria inoculation influences aphids and their natural enemies across the growing season. Inoculated barley plants generally showed reduced aphid population growth, though effects varied with barley variety, bacterial strain and aphid genotype. Suppression was strongest in glasshouse and choice experiments, whereas in outdoor pots, direct suppression was evident only in certain treatments later in the season. Notably, parasitoid wasps colonised inoculated plants earlier and in greater numbers, suggesting an indirect rhizobacteria‐mediated pathway through enhanced natural enemy recruitment. Variation in the headspace volatile profiles of inoculated plants, further modified by aphid presence, indicates that rhizobacteria can influence plant volatile emissions that may underlie this attraction. Together, these results show that rhizobacteria shape multitrophic interactions via both direct and indirect effects on herbivore populations.

Aphid suppression by rhizobacteria is mediated through induced host‐plant defences that reduce aphid longevity and fecundity.^19^, ^31^, ^32^ In the glasshouse, suppression was strongest during the vegetative and tillering stages, coinciding with peak aphid reproductive potential. As barley plants matured and senesced, *S. avenae* reproduction declined naturally,^33^ and rhizobacteria‐mediated protection was lost. Nevertheless, early suppression during aphid colonisation and population expansion had a lasting effect in the outdoor experiment with significant suppression on specific treatments later in the experiment. The glasshouse and host‐choice assays, conducted in controlled environments with confined aphids, mirror conditions used in previous studies.^34^, ^35^, ^36^, ^37^, ^38^ By contrast, aphids in the outdoor experiment encountered additional factors influencing population dynamics, including host‐finding via visual and olfactory cues,^39^ host acceptance through gustatory cues,^40^ and variable microclimates. Aphids on outdoor plants also experienced weather‐related mortality. Because pots were spaced by at least 0.5 m in the outdoor experiment, unwinged aphids likely remained on suboptimal hosts, while dislodged individuals had limited capacity to return. Thus, variation in suppression outdoors likely reflected combined effects of host resistance, dispersal behaviour, and environmental conditions.

In contrast to expectations, some inoculated plants in the outdoor experiment supported higher aphid numbers. We therefore hypothesised that aphids might be attracted to inoculated barley. However, a controlled host‐choice assay with unwinged aphids provided no evidence of a preference for inoculated plants; in fact, the pink aphids showed a slight preference against them. Aphid host‐choice is not always a direct reflection of plant quality but may depend on local aphid density, which influences population growth rates and the induction threshold for plant defences.^32^ High aphid density can also increase movement among plants and alter host‐choice.^41^ Furthermore, plant volatiles from the focal or a neighbouring plant can also influence aphid host‐choice,^42^ which means neighbouring inoculated plants may alter the response of the control plant (or *vice versa*) in a field population. Multiple aphid species (and likely genotypes) were present outdoors, which can further affect host preference.^43^ Consistent with earlier studies,^19^ we observed genotype‐specific responses: green aphids were suppressed by *B. subtilis* without showing host‐choice effects, while pink aphids were not suppressed but exhibited variable preferences. These findings suggest that rhizobacteria act through multiple, interacting mechanisms affecting aphid behaviour and performance. Overall, aphid abundance could not be explained by a single factor but instead reflected the combined influence of host resistance, aphid density, genotype and plant volatiles. Consequently, suppression effects are more readily detected in controlled environments, yet understanding their ecological complexity is essential for translating these findings to field systems.

In the outdoor pot experiment, parasitoid wasps arrived earlier on rhizobacteria‐inoculated barley, and parasitised aphid mummies were more abundant on these plants. The rhizobacteria effect was independent of aphid density, as shown by both additive and interactive models. Similar responses have been reported in Brassicaceae inoculated with *Bacillus* spp., including *B. subtilis*,^27^, ^28^ and with *Pseudomonas* spp. enhancing natural enemy recruitment.^25^, ^26^ We also observed early‐season recruitment with *A. radicis*, although this effect declined later in the season, suggesting possible seasonal or plant developmental influences that warrant further study.

Enhanced attraction of parasitoids to inoculated plants indicates a potential indirect, volatile‐mediated mechanism of plant protection. Rhizobacteria are known to modify plant biochemical pathways, including the emission of volatile organic compounds,^44^ which can influence both herbivores^45^ and their natural enemies.^46^, ^47^ Consistent with this, we found that rhizobacteria inoculation altered the volatile profile of barley, but that these effects were primarily due to changes in individual compounds rather than shifts across the entire blend (also see Parker^48^). This potentially explains the increased wasp attraction, and similar results have previously been shown in *Arabidopsis* and Brassicaceae.^23^, ^24^ Another possible mechanism is that rhizobacteria reduce aphid fitness or alter aphid physiology in ways that increase their susceptibility or detectability to parasitoids. Such changes may modify epicuticular cues used by wasps for host recognition.^49^ Because aphid density alone did not account for increased parasitoid abundance, we conclude that rhizobacteria mediate top‐down control of aphids through plant‐induced biochemical and behavioural mechanisms.

Many rhizobacteria are also considered plant‐growth promotors, yet there is a trade‐off for plants between growth and defence.^50^ We found minimal effects of rhizobacteria inoculation on plant growth during the season for the outside pot experiment. However, we did observe an increase in tiller number (seed heads) in *A. radicis*‐inoculated plants in the glasshouse experiment, which in a field situation would lead to a higher yield.^51^ However, the well‐studied *B. subtilis* seemed to reduce tiller number in our experiments. Although the rhizobacteria used in this study have all been considered plant‐growth promoting, these effects are often studied in controlled environments over shorter periods, especially for *A. radicis* and *P. simiae*.^35^, ^36^, ^52^ Vegetative growth for a grass such as barley does not necessarily correlate with increased yield, with modern varieties often bred to be shorter but with a higher yield.^53^ We used one heritage variety, Chevallier, in our experiments, in which we saw no aphid suppression and the lowest yield, despite producing the largest biomass, illustrating the trade‐off between growth and defence.

## 5 CONCLUSION

The three complementary experimental systems used in this study allowed us to disentangle how rhizobacterial inoculation influences aphid populations across different plant varieties, growth stages and environmental contexts. Although effects varied among systems, consistent patterns of aphid suppression revealed that rhizobacteria can modulate multiple interacting processes.^13^, ^54^ These include aphid host‐choice and settling behaviour, initiation of feeding and reproduction,^40^ plant susceptibility or resistance,^55^ and the recruitment of natural enemies.^46^ Our results suggest that rhizobacteria may influence each of these components to shape overall pest dynamics. In the outdoor pot experiment, where aphids were free to colonise plants naturally, population suppression was lower than in the glasshouse, likely reflecting the integration of both direct and indirect interactions under more variable conditions. This context dependency highlights the complexity of microbe‐mediated pest regulation but also its robustness across systems. Overall, rhizobacterial inoculation offers a promising complementary tool for pest management by enhancing host‐plant resistance and facilitating natural enemy activity. Future work should assess the consistency of these effects under field conditions, identify crop–microbe combinations that maximise pest suppression, and evaluate practical delivery methods for microbial inoculants within integrated pest management frameworks.

## CONFLICT OF INTEREST

The authors declare that there is no conflict of interest.

## Supporting information


**Figure S1.** Summary of experimental design.
**Fig. S2.** Summary of plant trait data.
**Fig. S3.** Summary of unwinged aphid species total data.
**Fig. S4.** Summary of *Rhopalosiphum padi* aphid data.
**Fig. S5.** Summary of *Metapolophium dirhodum* aphid data.
**Fig. S6.** Summary of yield data.
**Fig. S7.** Summary of aphid proportional data.

## Data Availability

Experimental data is available at http://doi.org/10.48420/30344521, with metabolomics data available through NERC EIDC. Rcode and data available via FigShare (https://doi.org/10.48420/30344521).
