# Supplementary material for: Rhizobacteria regulate colonising Sitobion avenae aphid populations through induced host resistance and alter plant volatiles promoting early parasitoid recruitment on barley ( Hordeum vulgare )
Source: Pest Manag Sci. 2026 Apr 10;82(7):7050–61. doi: 10.1002/ps.70783 (PMC13240696; doi:10.1002/ps.70783)
Supplement: Supplementary file 1 — Figure S1. Summary of experimental design. Fig. S2. Summary of plant trait data. Fig. S3. Summary of unwinged aphid species total data. Fig. S4. Summary of Rhopalosiphum padi aphid data. Fig. S5. Summary of Metapolophium dirhodum aphid data. Fig. S6. Summary of yield data. Fig. S7. Summary of aphid proportional data. [file PS-82-7050-s001.pdf]

ELECTRONIC SUPPLEMENTARY MATERIAL

Rhizobacteria regulate colonising *Sitobion avenae* aphid populations through induced host resistance and alter plant volatiles promoting early parasitoid recruitment on barley (*Hordeum vulgare*)

Megan E. Parker[1], Angharad M. R. Gatehouse[2] and Sharon E. Zytynska[1,†]

[1]Department of Evolution, Ecology and Behaviour, Institute of Infection, Veterinary and Ecological Sciences, University of Liverpool, L69 7ZB

[2]School of Natural and Environmental Sciences, Newcastle University, NE1 7RU

[†] current address: Department of Earth and Environmental Sciences, School of Natural Sciences, The University of Manchester, M13 9NT

Corresponding author: sharon.zytynska@manchester.ac.uk

Rcode and data available at http://doi.org/10.48420/30344521

Figure S1: Summary of experimental design

Figure S2: Summary of plant trait data

Figure S3: Summary of unwinged aphid species total data

Figure S4: Summary of *Rhopalosiphum padi* aphid data

Figure S5: Summary of *Metapolophium dirhodum* aphid data

Figure S6. Summary of yield data

Figure S7: Summary of aphid proportional data

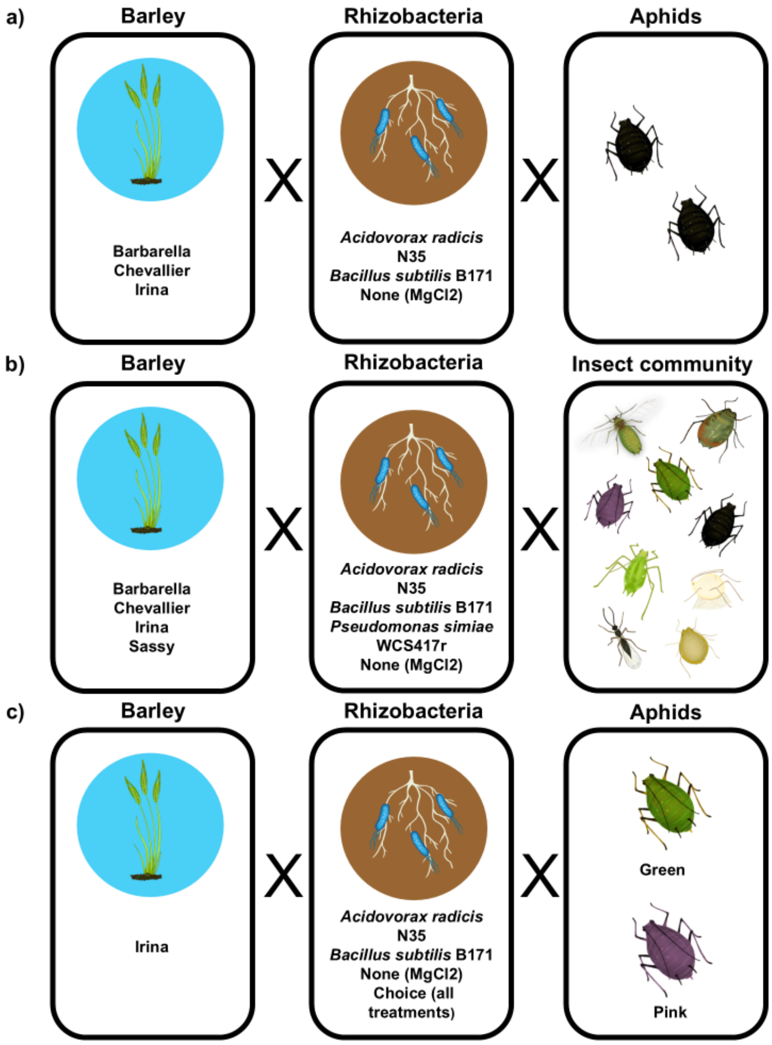

Figure S1: Summary of experimental design for (a) Experiment 1: glasshouse; (b) Experiment 2: outside pot; and (c) Experiment 3: aphid host-choice.

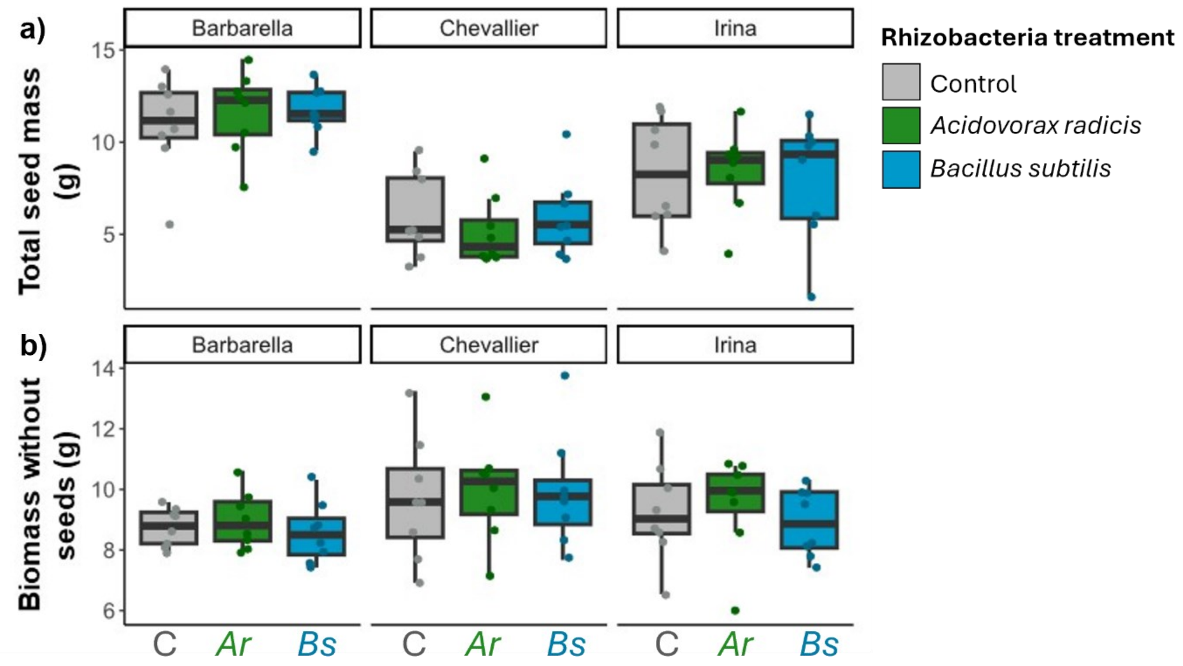

Figure S2: Summary of plant trait data from Experiment 1: glasshouse. a) Total seed mass per plant (g) and b) plant biomass without seeds (g) from barley (Barbarella, Chevallier and Irina) inoculated with Ar: *Acidovorax radicis* (green) and Bs: *Bacillus subtilis* (blue).

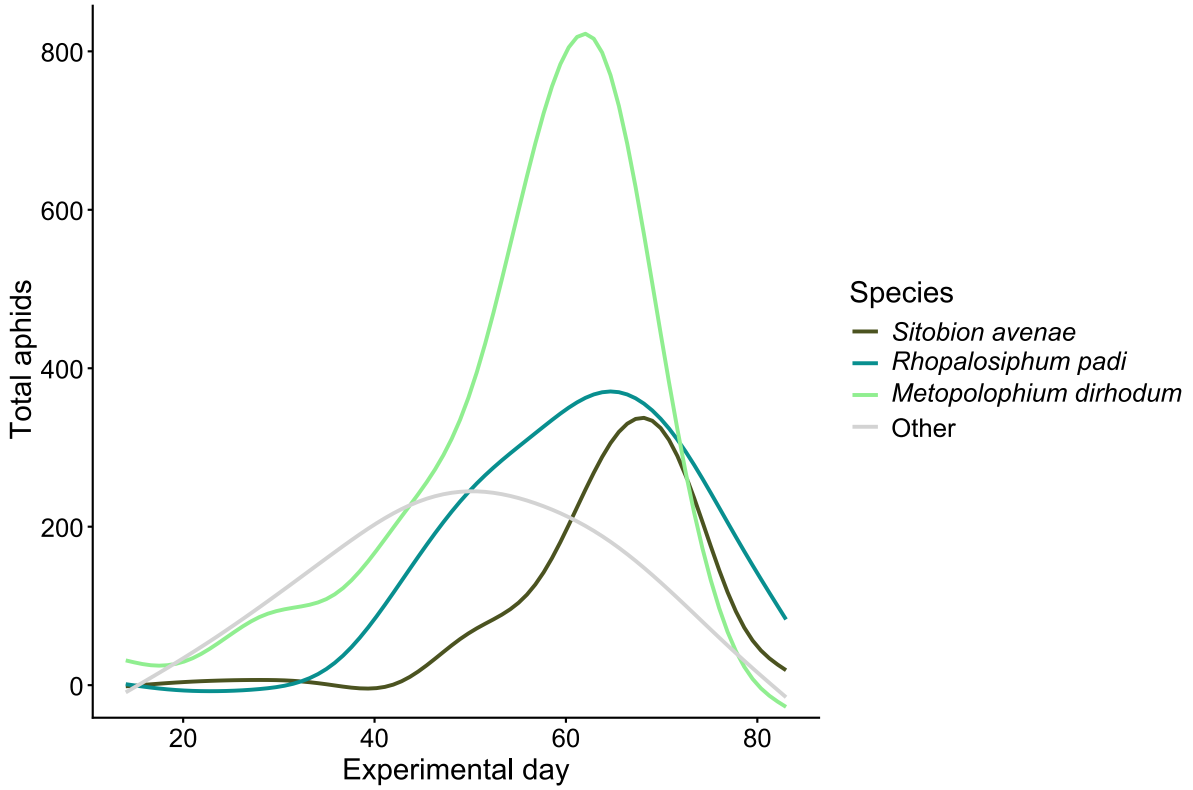

Figure S3: Summary of unwinged aphid species total data: Total unwinged aphids across all treatments across experimental duration separated by the species *Sitobion avenae*, *Rhopalosiphum padi*, *Metapolophium dirhodum* and other.

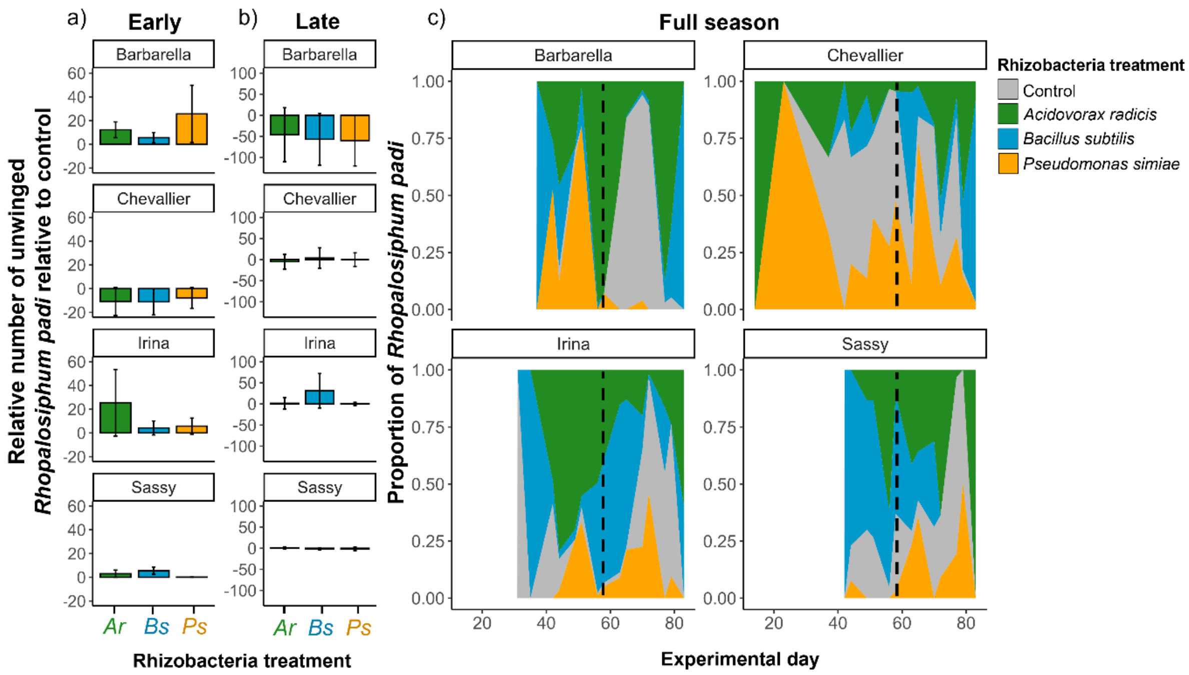

Figure S4. Unwinged *Rhopalosiphum padi* aphid data. Relative number of aphids compared to control plants (within block comparisons) for (a) early and (b) late season. Error bars show +/- 1SE. (c) Distribution of *R. padi* aphids across rhizobacteria treatments for each barley variety and across the experimental duration. Control: control treatment (grey), Ar: *Acidovorax radicis* (green), Bs: *Bacillus subtilis* (blue), Ps: *Pseudomonas simiae* (yellow). Dashed line shows split between early and late season for each barley variety. Empty space indicates no aphids found on plants of this variety

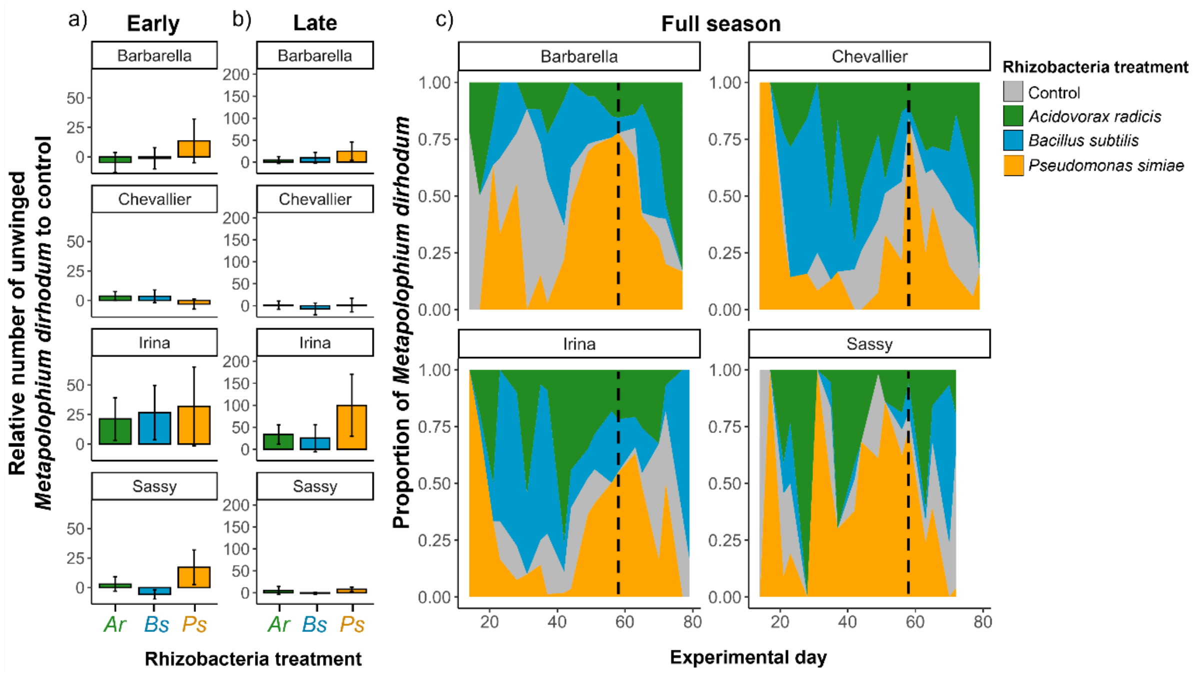

Figure S5. Unwinged *Metapolophium dirhodum* aphid data. Relative number of aphids compared to control plants (within block comparisons) for (a) early and (b) late season. Error bars show +/- 1SE. (c) Distribution of *M. dirhodum* aphids across rhizobacteria treatments for each barley variety and across the experimental duration. Control: control treatment (grey), Ar: *Acidovorax radicis* (green), Bs: *Bacillus subtilis* (blue), Ps: *Pseudomonas simiae* (yellow). Dashed line shows split between early and late season for each barley variety. Empty space indicates no aphids found on plants of this variety

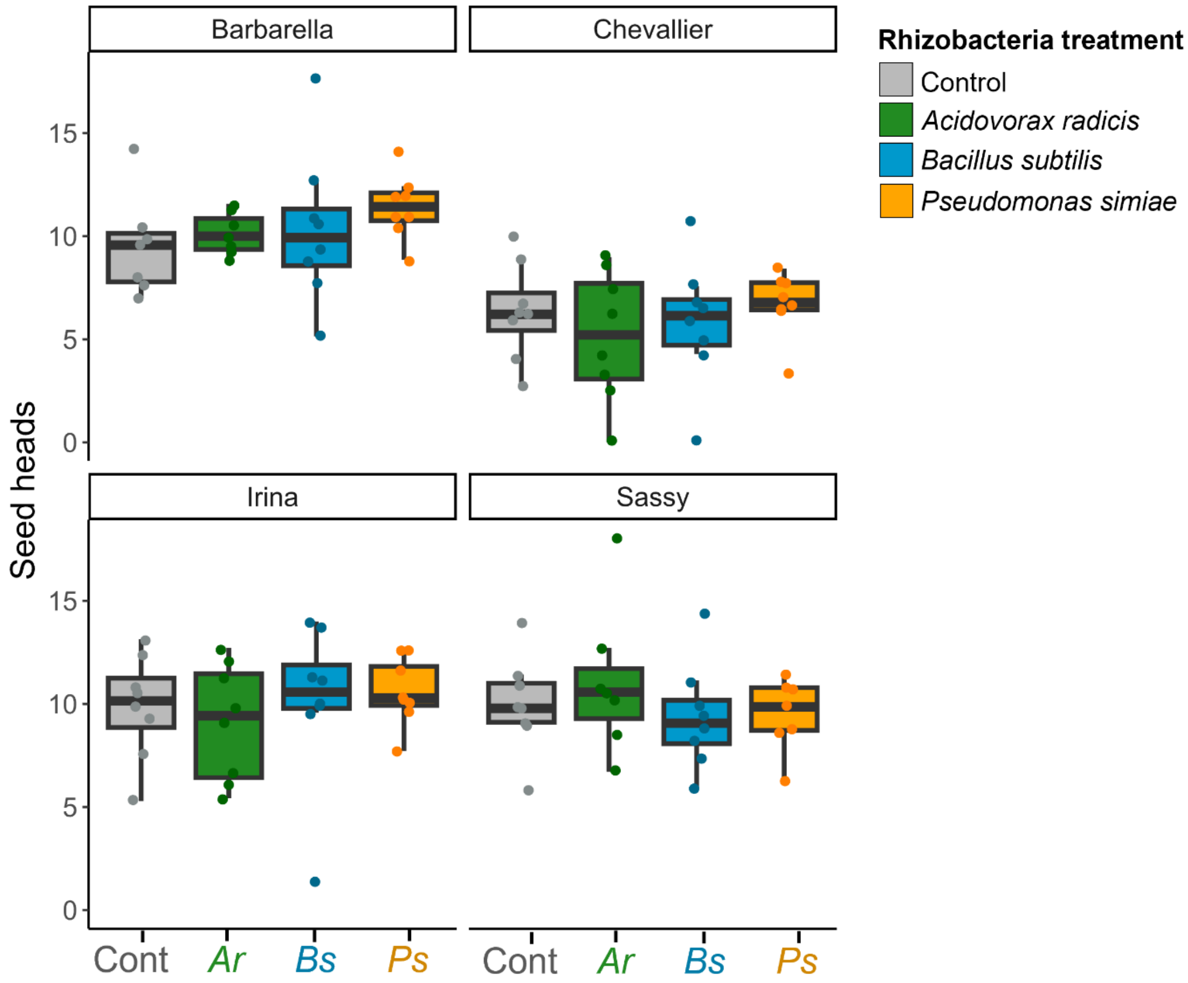

Figure S6. Summary of yield data. Final yield (total seed head count) for barley cultivars Barbarella, Chevallier, Irina and Sassy, rhizobacteria treatments: Cont: control treatment (grey), Ar: *Acidovorax radicis* (green), Bs: *Bacillus subtilis* (blue), Ps: *Pseudomonas simiae* (yellow).

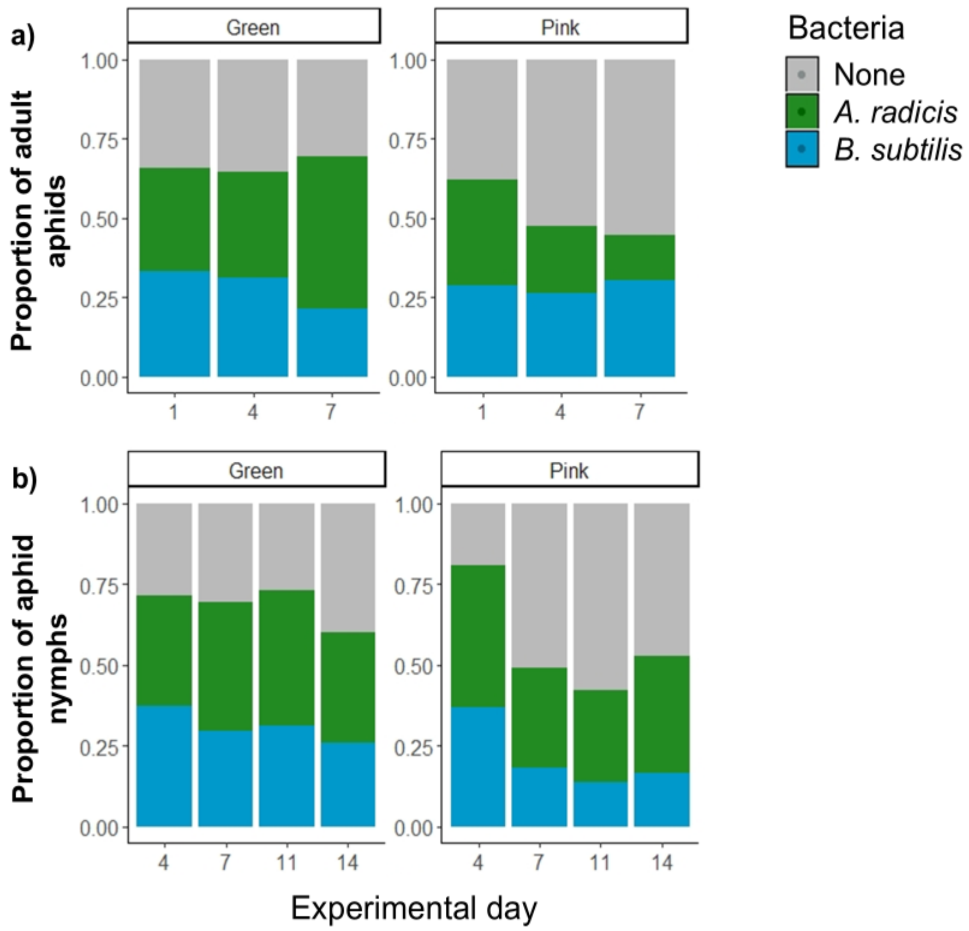

Figure S7. Aphid choice across all time points. (a) Proportion of adult aphids on each plant in choice pots (days 1, 4, 7), (b) Proportion of aphid offspring on each plant in choice pots (days 4, 7, 11, 14). Plants inoculated with control treatment (grey), Ar: *Acidovorax radicis* (green), Bs: *Bacillus subtilis* (blue).
